# Nanocellulose Sponges Containing Antibacterial Basil Extract

**DOI:** 10.3390/ijms241411871

**Published:** 2023-07-24

**Authors:** Gabriela Mădălina Oprică, Denis Mihaela Panaitescu, Catalina Diana Usurelu, George Mihai Vlăsceanu, Paul Octavian Stanescu, Brandusa Elena Lixandru, Valentin Vasile, Augusta Raluca Gabor, Cristian-Andi Nicolae, Marius Ghiurea, Adriana Nicoleta Frone

**Affiliations:** 1 National Institute for Research and Development in Chemistry and Petrochemistry, 202 Spl. Independentei, 060021 Bucharest, Romania; madalina.oprica@icechim.ro (G.M.O.); catalina.usurelu@icechim.ro (C.D.U.); raluca.gabor@icechim.ro (A.R.G.); cristian.nicolae@icechim.ro (C.-A.N.); ghiurea@gmail.com (M.G.); 2 Faculty of Chemical Engineering and Biotechnology, University Politehnica of Bucharest, 1-7 Gh. Polizu Street, 011061 Bucharest, Romania; george.vlasceanu@upb.ro (G.M.V.); paul.stanescu@upb.ro (P.O.S.); 3 Cantacuzino National Medical-Military Institute for Research and Development, 103 Spl. Independentei, 050096 Bucharest, Romania; brandusa_lixandru@yahoo.com (B.E.L.); vali909.g@gmail.com (V.V.)

**Keywords:** cellulose, basil extract, antibacterial, nanofibers, microstructure, porosity

## Abstract

Nanocellulose (NC) is a valuable material in tissue engineering, wound dressing, and drug delivery, but its lack of antimicrobial activity is a major drawback for these applications. In this work, basil ethanolic extract (BE) and basil seed mucilage (BSM) were used to endow nanocellulose with antibacterial activity. NC/BE and NC/BE/BSM sponges were obtained from nanocellulose suspensions and different amounts of BE and BSM after freeze-drying. Regardless of the BE or BSM content, the sponges started to decompose at a lower temperature due to the presence of highly volatile active compounds in BE. A SEM investigation revealed an opened-cell structure and nanofibrillar morphology for all the sponges, while highly impregnated nanofibers were observed by SEM in NC/BE sponges with higher amounts of BE. A quantitative evaluation of the porous morphology by microcomputer tomography showed that the open porosity of the sponges varied between 70% and 82%, being lower in the sponges with higher BE/BSM content due to the impregnation of cellulose nanofibers with BE/BSM, which led to smaller pores. The addition of BE increased the specific compression strength of the NC/BE sponges, with a higher amount of BE having a stronger effect. A slight inhibition of *S. aureus* growth was observed in the NC/BE sponges with a higher amount of BE, and no effect was observed in the unmodified NC. In addition, the NC/BE sponge with the highest amount of BE and the best antibacterial effect in the series showed no cytotoxic effect and did not interfere with the normal development of the L929 cell line, similar to the unmodified NC. This work uses a simple, straightforward method to obtain highly porous nanocellulose structures containing antibacterial basil extract for use in biomedical applications.

## 1. Introduction

Cellulose, one of the most abundant polysaccharides on earth, is a valuable material in tissue engineering, wound dressing, and drug delivery, bringing to the table its low toxicity, biodegradability, and biocompatibility [1,2]. Its nano-version is in high demand in the medical field due to its high surface area and the capacity to undergo extensive physical and chemical modifications that enhance its properties [3,4]. Nanocellulose (NC) is obtained from a multitude of natural resources by mechanical, chemical, enzymatic, or combined treatments, and it is also synthesized by bacteria [5]. The porous nature of nanocellulose sponges strongly supports their ability to absorb active compounds or exudates that are present in infected tissues, control the moisture of the affected area, and transport or release active principles [3,6,7]. A spin-assisted layer-by-layer assembly method was used to make multi-layered patches composed of chitosan, alginate, and bacterial cellulose (BC) layers [6]. Developed for wound healing applications, these patches contained dexpanthenol, a precursor of pantothenic acid, impregnated into the BC layer, serving as a drug reservoir. The patches managed to stop the growth of *Staphylococcus aureus* and were non-cytotoxic to human keratinocytes [6]. Similarly, BC membranes impregnated with aqueous solutions of hyaluronic acid and diclofenac showed controlled release of the active compounds and promising results in treating aphthous stomatitis [2].

Many studies have been focused on the synthesis of nanocellulose sponges or films with antimicrobial activity [8,9,10]. The lack of antimicrobial activity is a major drawback to otherwise successful nanocellulose applications in the biomedical and packaging fields. Several methods have been studied so far for endowing nanocellulose with antibacterial properties: surface modification of NC with aldehyde or quaternary ammonium groups, addition to NC of metal or metal oxide nanoparticles, and incorporation of antibiotics or antibacterial agents [3,9], among others. In particular, coating NC with copper–copper oxide nanoparticles using an alcoholic extract of *Terminalia chebula* fruit as a reducing agent [11], immersing BC film in a zinc salt for incorporating ZnO nanoparticles [12], ZnO plasma coating of a polyhydroxybutyrate/bacterial cellulose nanofiber film [13], incorporating NC and TiO_2_ in wheat gluten [14], or the synthesis of silver nanoparticles on a NC substrate [15] led to NC materials showing different degrees of antibacterial activity against Gram-positive and Gram-negative bacteria. However, these popular antibacterial agents have different levels of toxicity based on their concentration, and it has been reported that bacteria have become resistant to heavy metals [16]. Moreover, the use of antibiotics is restricted by the increasing inefficiency of such conventional treatments due to antibiotic-resistant bacteria [17]. Therefore, finding effective antibacterial agents is a prominent challenge on the current research agenda.

Fortunately, nature gives us endless opportunities to find compounds that, besides antibacterial activity, combine desirable properties such as low toxicity, biodegradability, and biocompatibility. Multiple approaches for obtaining antibacterial nanocellulose using various plant extracts and oils have been reported [16,18]. In particular, curcumin, a natural product extracted from *Curcuma longa* (turmeric), was incorporated into a NC/polyvinyl alcohol (PVA) matrix, and the mixture was cast as a film intended for diabetic wound dressing application [16]. The obtained film showed inhibitory activity on three Gram-positive bacteria, including methicillin-resistant *Staphylococcus aureus*. In another study, BC impregnated with an ethanolic extract of mangosteen (*Garcinia mangostana*) showed a strong inhibitory effect on *Staphylococcus epidermidis*, *Propionibacterium acnes*, and *Staphylococcus aureus* due to the polyphenolic compounds contained in the mangosteen extract [18].

Basil (*Ocimum Basilicum* L.) belongs to the *Lamiaceae* family and is rich in valuable compounds for biomedical applications [19]. Known to everyone as a culinary aid and a traditional medicine used to treat headaches, colds, inflammatory diseases, and others [20], basil contains polyphenols that brought it into the scientific limelight. However, the chemical composition of this plant varies based on the geographical position and the variety of basil considered [21,22], but generally speaking, every variety contains phenolic compounds, which are the basis for their antibacterial, hypoglycemic, and antioxidant activity [23]. The study of the chemical composition of basil showed several types, one abundant in linalool (at least 50%), a monoterpene alcohol that is mostly found in Europe; another type rich in methyl cinnamate (50–70%) besides linalool and camphor, which is popular in tropical regions; and others with high concentrations of methyl chavicol or eugenol [21,22].

Considering its important properties, basil has been used as an active compound in several biopolymers to obtain materials characterized by antioxidant, antibacterial, and anticancer properties for packaging and biomedical applications [24,25]. Nanocomposite films containing PVA, cellulose nanocrystals (CNCs), and different amounts of an aqueous extract of basil leaves were obtained using the solution casting method. Only the nanocomposite with the largest concentration of basil leaf extract (20% *wt/v*) showed measurable antibacterial activity against *Bacillus cereus* [24]. Similarly, an aqueous extract of basil leaves was added to a chitosan/TEMPO (2,2,6,6-tetramethylpiperidine-1-oxy)-oxidized cellulose fibers (2:1) mixture, and the new material was characterized to evaluate its applicability in food packaging, wound healing, or pharmaceutics [25]. The composite containing basil extract acted as a strong inhibitor for the Gram-positive strains (*Streptococcus mutans*) but was less efficient for the Gram-negative ones (*Salmonella typhimurium*). The synergistic effect of the phenolic groups of the basil extract and the functional groups (amino or carboxyl) of the composite were also considered responsible for the antibacterial activity [25]. Cassava starch/nanocrystalline cellulose (6 wt%) films enriched with the ethanolic extract of basil leaves (from 2 to 10% *v*/*v*) showed a decrease in tensile strength and an increase in elongation at break with the increase in the content of basil extract due to the plasticizing effect of the extract and good antioxidant activity [26]. Several studies have shown the antioxidant effect of basil essential oil (BEO) in chitosan/halloysite nanotubes/BEO films [27] or polyethylene/chitosan/BEO films [28] intended for active packaging applications. The antimicrobial activity of BEO as such, microencapsulated, or as chitosan films containing BEO microcapsules for food packaging has also been demonstrated against Gram-positive and Gram-negative bacteria [29].

The mucilage obtained from basil seeds drenched in water has also been studied as an additive or matrix in drug delivery systems [19], in food packaging [30], and for other applications in the food industry [31,32]. Basil seed mucilage (BSM) has a heteropolysaccharide structure consisting of glucomannan and (1,4)-linked xylan, along with minor amounts of uronic acid [33,34]. BSM has low toxicity and high viscosity with pseudoplastic character and is being studied as a novel hydrocolloid in the food industry to improve texture and resistance to aging, stabilize emulsions, and enhance water retention [33,34,35]. However, following a survey of the existing literature, no study on the basil extract’s ability to endow nanocellulose sponges with antimicrobial properties while preserving their biocompatibility for wound healing applications has been reported so far.

**Figure 1:**
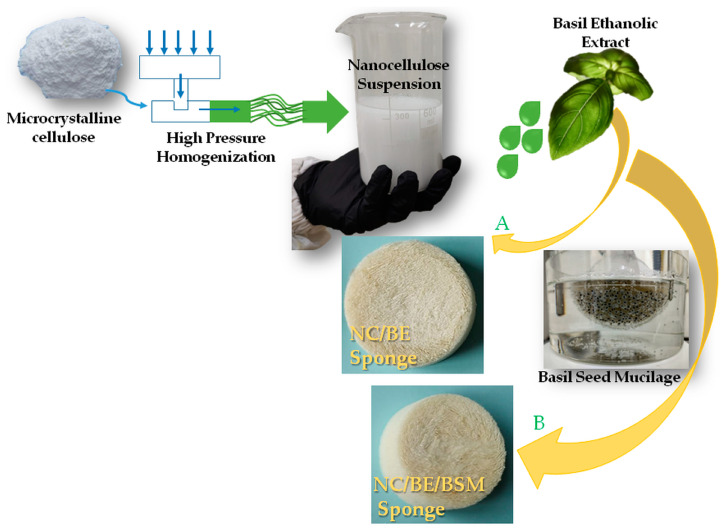
Schematic representation of the synthesis of NC/BE and NC/BE/BSM sponges.

In this study, NC sponges with antibacterial activity were obtained from nanocellulose suspensions and different amounts of basil ethanolic extract (BE) after freeze-drying. Basil seed mucilage was also used to improve the properties of the sponges. Figure 1 shows a schematic representation of the synthesis of the NC/BE and NC/BE/BSM sponges. The morpho-structural characteristics and thermal and mechanical properties were investigated using Fourier-transform infrared spectroscopy (FT-IR), scanning electron microscopy (SEM), microcomputer tomography (micro-CT), thermogravimetric analysis (TGA), and dynamic mechanical analysis (DMA). An in vitro study to assess the antibacterial activity and cytotoxicity of selected sponges was carried out as well. This work uses a simple, straightforward method with the ultimate purpose of obtaining antibacterial and biocompatible, highly porous structures for biomedical applications.

## 2. Results

### 2.1. FT-IR Spectroscopy Analysis

**Figure 2:**
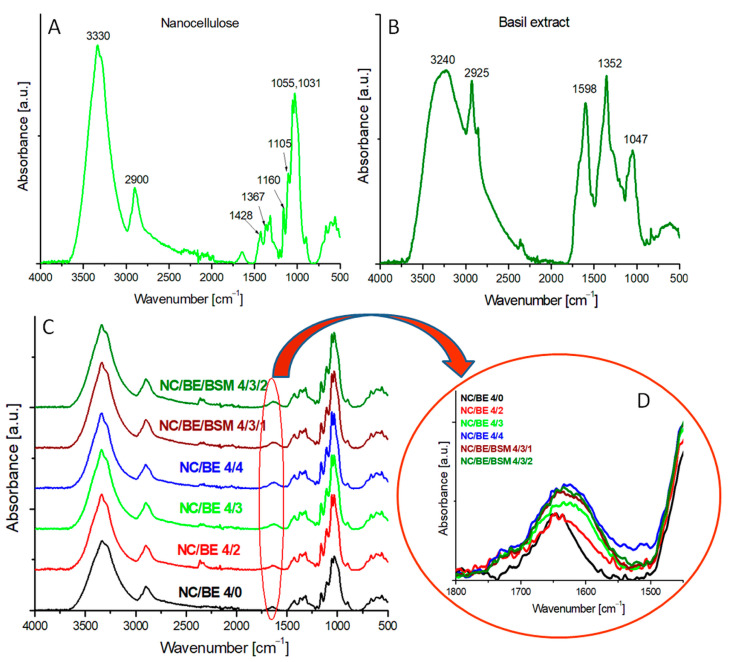
FT-IR spectra of nanocellulose—NC (**A**), basil extract—BE (**B**), and nanocellulose sponges with different proportions of BE or BE/basil mucilage (BSM) (**C**); FT-IR spectra in the region of 1800–1450 cm^−1^ (**D**).

The FT-IR spectrum of pristine nanocellulose (NC/BE 4/0) (Figure 2A) has a broad band between 3650 and 3000 cm^−1^, associated to the stretching vibrations of hydrogen-bonded hydroxyl groups, abundant on the cellulose’s surface; a well-defined band at about 2900 cm^−1^, which is attributed to CH_2_ stretching vibrations; a small peak at about 1640 cm^−1^, which is generally assigned to the bending vibrations in bound water and to carboxylates resulting from the oxidation of cellulose; smaller peaks at 1428 and 1367 cm^−1^, which are related to CH wagging and bending, respectively; and important vibrations at 1160, 1105, 1055, and 1031 cm^−1^, which are assigned to C–O–C asymmetrical bridge stretching, C–O/C–O–C stretching, C–O–C pyranose ring stretching, and C–O/C–C stretching, respectively [36,37,38,39]. A distinctive peak found at 899 cm^−1^ corresponds to the β-glycosidic linkages in the glucose units.

The FT-IR spectrum of basil extract (Figure 2B) has a broad peak at 3240 cm^−1^, which is assigned to the stretching mode of hydrogen-bonded hydroxyl groups in alcohols, phenols, and carboxylic acids found in the composition of basil; sharp peaks at 2925 and 2851 cm^−1^, which are related to alkane C–H; and peaks at 1598, 1352, and 1047 cm^−1^, which are attributed, respectively, to C=C double bonds, alkane C–H, and C–O stretching in alcohols, ethers, and carboxylic acids [33,39,40,41]. Signals at 777, 828, and 874 cm^−1^ are associated with ring vibrations. A shoulder is observed at around 1700 cm^−1^, ascribed to the stretching vibrations of C=O groups from proteins (because basil extract also contains proteins in small amounts) [33,42].

The FT-IR spectra of all the mixtures are similar and contain the same bands, all of which are also characteristic of NC. This is not surprising since BE contains polysaccharides and its FT-IR spectrum shows many peaks similar to those of NC. However, the detailed FT-IR spectra of the sponges in the region from 1800 to 1500 cm^−1^ (Figure 2D) show that the band centered at 1640 cm^−1^ (corresponding to the absorbed water in unmodified NC) is much broader and skewed toward lower wavenumbers in the sponges containing BE or BS/BSM. This effect is enhanced in the sponges containing an increased amount of BE, with the half-width at half-maximum of the peaks increasing with the BE concentration in the sponges. The analysis of the deconvoluted FT-IR spectra using the Voigt model (Figure S1—Supplementary Material) shows new peaks at 1599 and 1720 cm^−1^ in the deconvoluted spectrum of NC/BE 4/3 as compared to that of NC/BE 4/0 (unmodified NC). These peaks can be assigned, respectively, to the C=C double bonds and C=O groups contained in the basil extract [40,43]. When comparing both sets of sponges, with and without the mucilage, a similarity was observed between them, as no evident wavenumber shifting was registered. For both sets, an increase in the intensity and broadening of the peak at around 1650 cm^−1^ was noticed.

### 2.2. Thermogravimetric Analysis (TGA)

**Figure 3:**
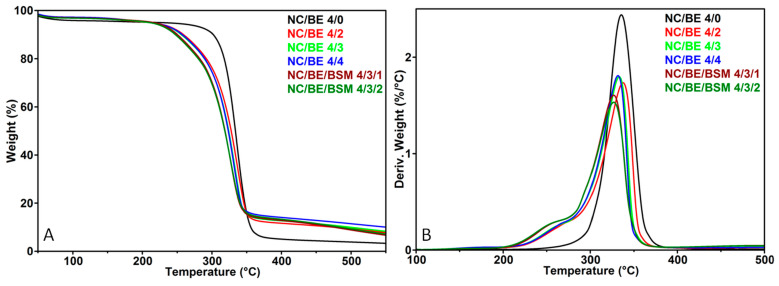
TG (**A**) and DTG (**B**) curves of unmodified NC (NC/BE 4/0), NC/BE, and NC/BE/BSM sponges.

The influence of BE and BE/BSM on the thermal stability of nanocellulose sponges was investigated by TGA, with the TG and derivative TG (DTG) curves shown in Figure 3. The most relevant characteristics, i.e., the temperature at 5% weight loss (T_5%_), the onset degradation temperature (T_on_), the temperature at the maximum degradation rate (T_max_), and the residue at 600 °C (R_600 °C_), were calculated from the thermograms and are shown in Table 1.

The slight weight loss registered for all the sponges between 50 and 150 °C was generally attributed to the vaporization of the moisture trapped in the sponges. The major weight loss observed between 250 and 400 °C was caused by the dehydration and depolymerization reactions through the breaking off of glycosidic bonds within the cellulose chain [44]. All the sponges containing BE or BE/BSM presented the beginning of the thermal degradation at a lower temperature in the TG diagrams (Figure 3A) and a shoulder before the main degradation peak of cellulose in the DTG diagrams (Figure 3B). This shift in the T_5%_ and T_on_ of less than 10 and 25 °C, respectively (Table 1), and the presence of the shoulder centered at 250 °C were due to the BE component, whose active compounds suffer thermal decomposition starting at a lower temperature (230 °C [45]) than cellulose (315 °C—Table 1). Linalool, methyl chavicol, methyl cinnamate, and eugenol, found in different proportions in BE, are characterized by high volatility as they evaporate at a lower temperature [45]. Therefore, a small variation in the T_max_ (332–335 °C) was observed in the NC/BE sponges, regardless of the concentration of BE, due to the evaporation of the main components of the basil extract up to 330 °C [44]. The NC/BE/BSM sponges showed the highest decrease in thermal stability (Figure 3) due to the increase in highly volatile compounds. For the NC/BE sponges, the residue at 600 °C increased with the increase in BE content due to the formation of a high amount of char [44]. Interestingly, the thermal stability of NC/BE sponges, considering the T_max_ values, was better or similar to that of other nanocellulose sponges [6,44,46].

It should be remarked that the presence of basil extract or, additionally, basil seed mucilage in nanocellulose has only a slight influence on the thermal stability of the nanocellulosic sponges. Considering their intended use in biomedical applications, the thermal stability of NC/BE and NC/BE/BSM sponges is excellent at body temperature, where the volatile compounds in basil extract are still stable.

### 2.3. Scanning Electron Microscopy (SEM) Analysis

**Figure 4:**
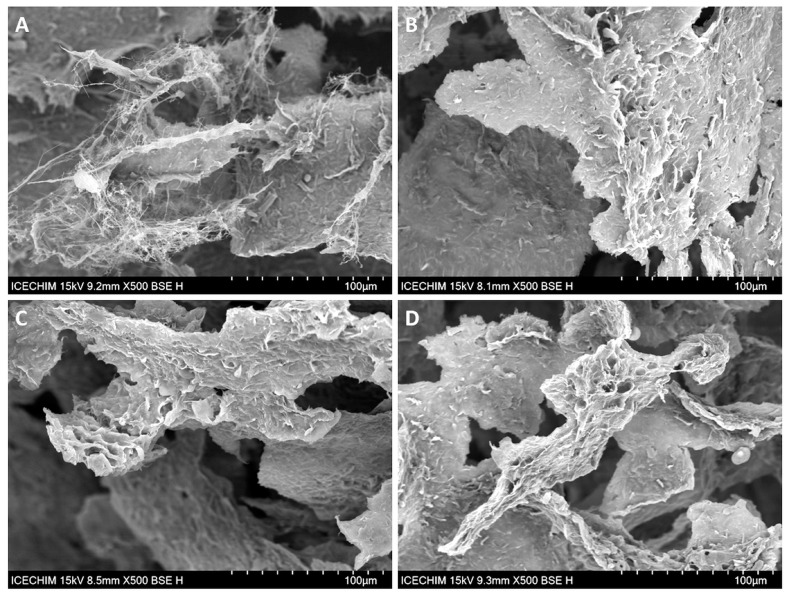
SEM images of NC/BE sponges, magnification ×500: NC/BE 4/0 (**A**); NC/BE 4/2 (**B**); NC/BE 4/3 (**C**); NC/BE 4/4 (**D**).

**Figure 5:**
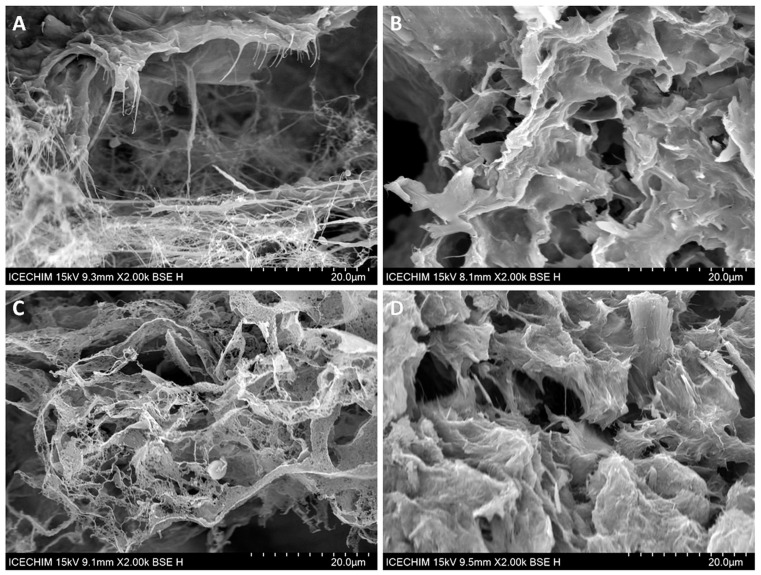
SEM images of NC/BE and NC/BE/BSM sponges, magnification ×2000: NC/BE 4/0 (**A**); NC/BE 4/3 (**B**); NC/BE/BSM 4/3/2 (**C**); NC/BE 4/4 (**D**).

The SEM analysis of the cross-sections of NC sponges containing different proportions of BE are shown in Figure 4. Large pores delimited by NC “walls” formed by ice sublimation during the freeze-drying process can be observed in the SEM images of all the sponges. The SEM images show that all the sponges have a large surface area due to the spaces delimited by “walls” but also to their microstructure, which offers multiple possibilities for incorporating active compounds. This microstructure is better observed in the SEM images with higher magnification (Figure 5). The presence of BE led to a more compact structure of the sponges (Figure 5B,D) due to impregnation of NC with BE and reduced zones with free cellulose nanofibers, which are frequently observed in unmodified NC sponges (Figure 5A—NC/BE 4/0). A similar effect was observed in the case of bacterial cellulose sponges after incorporating the bioactive icariin-complexed β-cyclodextrin [47].

These SEM images with higher magnification (Figure 5) show an opened-cell structure and a nanofibrillar morphology for all the sponges. The SEM image of the unmodified NC (Figure 5A) shows a fine network of cellulose nanofibers forming “walls”, while the SEM images of the NC/BE 4/3 (Figure 5B) and NC/BE 4/4 sponges (Figure 5D) show highly impregnated nanofibers. The addition of mucilage led to a more porous structure and a different organization of the BE/BSM “coated” nanofibers, which form a “lace-like” structure (Figure 5C—NC/BE/BSM 4/3/2). A similar structure was observed in surface-modified bacterial cellulose sponges crosslinked with citric acid [48].

Although the SEM analysis highlighted the opened porous structure of NC/BE 4/0, NC/BE 4/3, NC/BE/BSM 4/3/1, and NC/BE 4/4 sponges and the morphological features at the micro and nano levels, a quantitative evaluation of the pores and walls was not possible by SEM. Therefore, microcomputer tomography measurements were undertaken on these samples, and the results are discussed below.

### 2.4. Microcomputer Tomography (Micro-CT)

**Figure 6:**
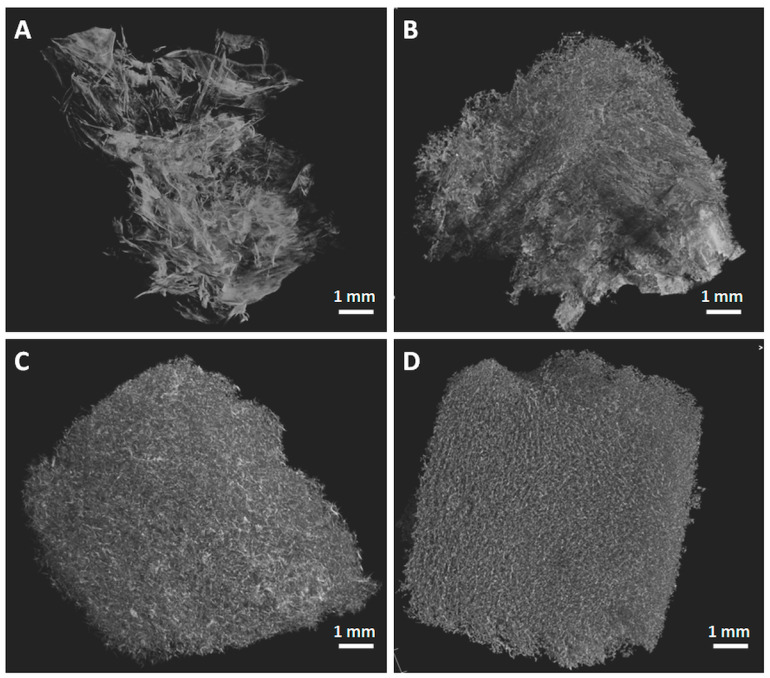
Micro-CT images of nanocellulose sponges containing different amounts of BE: BE 4/0 (**A**); BE 4/3 (**B**); BE 4/3/2 (**C**); BE 4/4 (**D**).

Micro-CT presents several advantages over SEM because it allows for the investigation of the tridimensional morphology of the sponges without the need for sectioning and gives important information about the void type and porosity [49]. The porous structure of the selected sponges is shown in Figure 6. The different electronic densities for nanocellulose fibers and pores determined a lighter color for the first ones. All the sponges exhibited an open porous morphology, with cellulose nanofibers forming “walls” and interconnected pores. Unmodified NC (NC/BE 4/0) has a laminar architecture with cellulose nanofibers and pores separated by large voids (Figure 6A). A different morphology with a higher degree of organization was noticed for the NC/BE sponges, which also preserved the high open porosity. The open porosity of the sponges was 82% for unmodified NC, 77% for NC/BE 4/3, 70% for NC/BE/BSM 4/3/2, and 72% for NC/BE 4/4. These values highlight the effect of BE and BSM, impregnated into the cellulose nanofiber network, leading to smaller pores. This observation is also supported by the lower average pore size obtained for the sponges containing a higher proportion of natural modifiers, such as NC/BE/BSM 4/3/2 and NC/BE 4/4 (99 and 108 µm, respectively), when compared to the sponges without BE (NC/BE 4/0) or those with lower BE concentration (NC/BE 4/3), with pore sizes of 261 and 170 µm, respectively. Similar average pore sizes (191 and 239 µm) were obtained by micro-CT for nanocellulose sponges containing 50/50 and 75/25 anionic/cationic cellulose nanofibers [50], respectively. Remarkably, the specific surface area, illustrated by the surface/volume calculation, was only slightly modified by the addition of BE or BE/BSM in the nanocellulose, from 60.2 µm^−1^ for NC/BE 4/0 (unmodified NC) to 57.1 µm^−1^ for NC/BE 4/3, to 64.8 µm^−1^ for NC/BE/BSM 4/3/2, and to 64.6 µm^−1^ for NC/BE 4/4.

**Figure 7:**
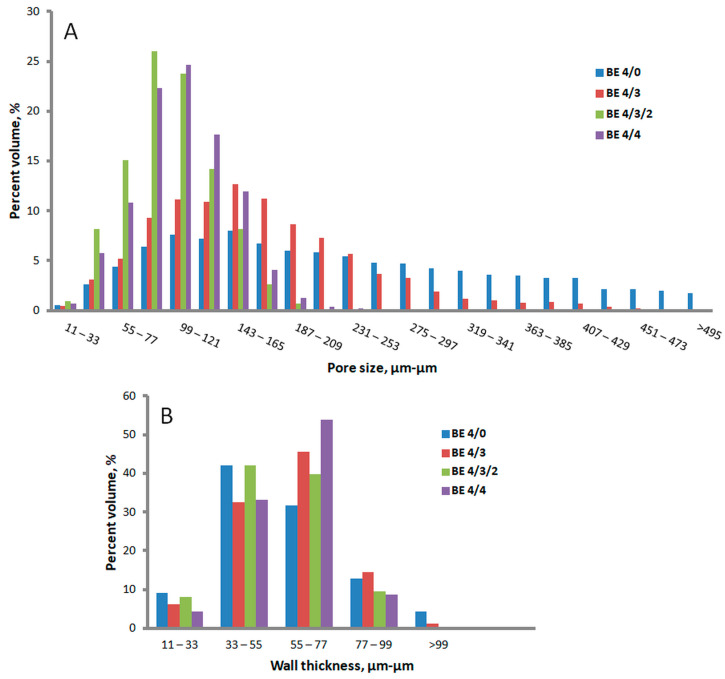
Micro-CT analysis: pore-size (**A**) and wall-thickness (**B**) distributions.

A deeper analysis of the pore-size and wall-thickness distributions for the analyzed sponges is presented in Figure 7. The unmodified NC sponge showed the broadest range of pore-size distribution, with very large pores (50.6% with more than 200 µm in diameter) being predominant. The proportion of pores exceeding 200 µm was lower for NC/BE 4/3 (27.3%) and much reduced for the sponges modified with large concentrations of BE and BSM (0.2% for NC/BE/BSM 4/3/2 and 0.7% for NC/BE 4/4). The wall-thickness distribution was not significantly influenced by the presence of BE or BE/BSM in the sponges or their concentration, showing that the natural products impregnated the fibers, not the walls.

### 2.5. Dynamic Mechanical Analysis (DMA)

**Figure 8:**
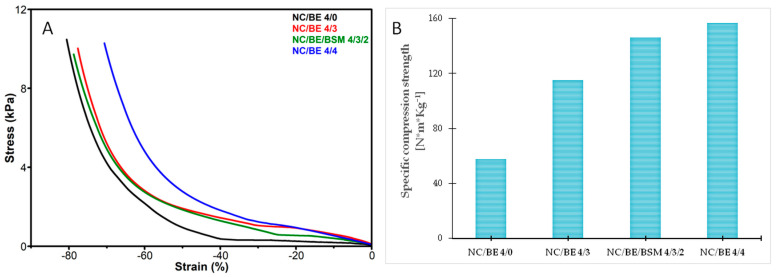
Compression stress—strain curves of NC, NC/BE, and NC/BE/BSM sponges (**A**); specific compression strength at 50% strain (**B**).

The stress—strain curves obtained following the dynamic mechanical analysis of the selected sponges and unmodified nanocellulose are presented in Figure 8A. Among all the sponges, NC/BE 4/4 exhibited the best stress/strain response, better supporting a higher force than the unmodified NC or the NC modified with a lower BE concentration. The elasticity of the sponges was not significantly improved with the addition of BSM (Figure 8A), although the potential of BSM to increase the flexibility and processability of different matrices was previously reported: the addition of BSM increased the elasticity of a whey protein isolate emulsion-filled gel [51] and improved the electrospinning ability of whey protein isolate [52]. However, the role of BSM in improving the tridimensional structure of the NC/BE sponges was highlighted by micro-CT (Figure 6).

The influence of each additive on the mechanical properties of nanocellulose sponges is highlighted in Table 2, which summarizes the apparent density and the compression strength at 50% strain for all the sponges. The increase in the quantity of BE in the NC sponges led to a continuous rise in their compression strength, with the NC/BE 4/4 sponge with the largest content of BE showing a three-time higher compression strength compared to that of the unmodified NC. Conversely, the addition of BSM to the NC/BE 4/3 sponge had no evident effect on its compression strength.

It can be observed that the NC/BE 4/4, which exhibited the best mechanical properties, also showed the highest apparent density (Table 2). An increase in the density of sponges is generally linked to an enhancement in their mechanical properties [48]. Therefore, the specific compression strength was calculated, and the results are presented in Figure 8B. It is obvious that the addition of BE increased the specific compression strength of the NC/BE sponges, with a higher amount of BE having a stronger effect, while the concomitant addition of BE and BSM further improved the specific compression strength. This may be explained by the better organization observed in the tomographs of these samples (Figure 6).

### 2.6. X-ray Diffraction (XRD)

**Figure 9:**
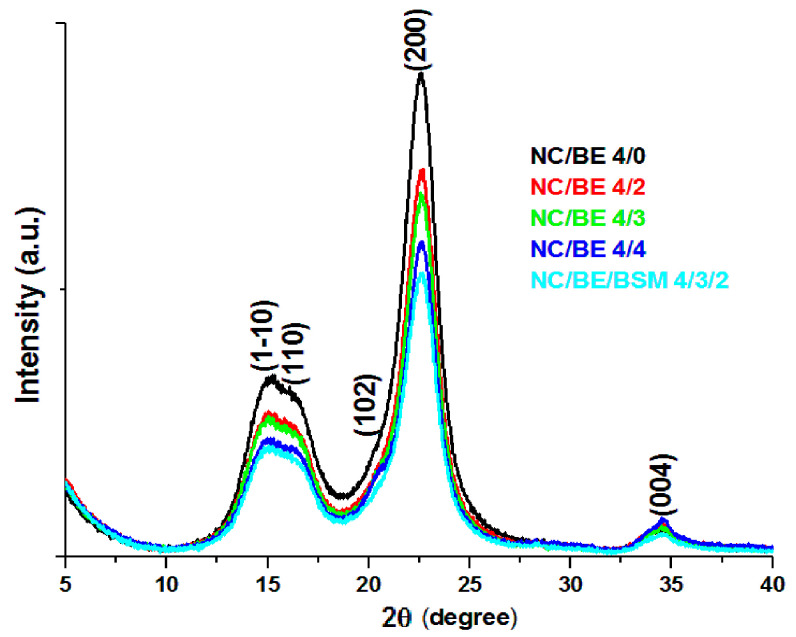
XRD patterns of NC (NC/BE 4/0), NC/BE (NC/BE 4/2, NC/BE 4/3, and NC/BE 4/4), and NC/BE/BSM 4/3/2 sponges.

The XRD patterns of the nanocellulose sponges are shown in Figure 9. The addition of BE or BE/BSM to nanocellulose had no influence on its crystalline structure. Three main peaks appeared at 2θ 14.8°, 16.4°, and 22.6°, and they correspond to the diffraction of the (1–10), (110), and (200) planes of Iβ cellulose crystal structure [53,54], respectively. A shoulder at about 21° and a small peak at 34.5°, corresponding to lattice planes (102) and (004), respectively, were also observed (Figure 9). These peaks appeared more clearly after deconvolution (Figure S2—Supplementary Material). Each pattern was deconvoluted into Voigt components by using Fityk 1.3.1 software [55], and the crystallinity index (CI) was calculated as the ratio between the area under the crystalline peaks and the sum of all the peak areas [54]. Small differences were observed between the CI values of the unmodified NC (77%) and those of the NC/BE sponges, i.e., 78% for NC/BE 4/2 and NC/BE 4/3, 77% for NC/BE 4/4, and 75% for NC/BE/BSM 4/3/2. These results show a high degree of crystallinity for all the sponges, similar to the CI of MCC [56] and the CI values reported for other nanocellulose products [46,57].

### 2.7. Antibacterial Activity

**Figure 10:**
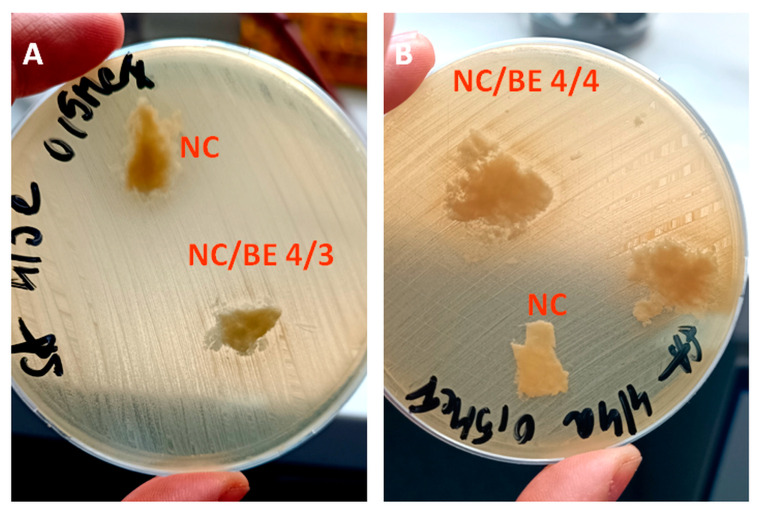
Antibacterial activity of NC/BE 4/3 (**A**) and NC/BE 4/4 (**B**) against *Staphylococcus aureus*.

The disc diffusion method was used to evaluate the antibacterial activity of the sponges against *Staphylococcus aureus* and *Escherichia coli*. A slight inhibition of *S. aureus* growth was observed in the NC/BE 4/3 and NC/BE 4/4 sponges, and no effect was observed in the unmodified nanocellulose (NC). An inhibition zone of about 2 mm can be seen in Figure 10 near the NC/BE 4/3 and NC/BE 4/4 sponges, showing their slight antibacterial activity. No growth inhibition of *E. coli* was observed, regardless of the concentration of BE in the sponges. This different effect is determined by the structure of the bacterial cell walls of the two types of bacteria: *S. aureus* bacteria have a single cytoplasmic membrane (as do all Gram-positive bacteria) that is covered with a thick peptidoglycan coating, while *E. coli* bacteria have an outer lipid membrane and a plasma membrane covered with a thin layer of peptidoglycan [30]. Therefore, the components of BE will more easily damage the membrane of Gram-positive bacteria, in agreement with our results. A similar slight bacteriostatic effect against *S. aureus* was reported for conjugated basil seed gum and alginate matrix [58].

### 2.8. Cytotoxicity Tests

**Figure 11:**
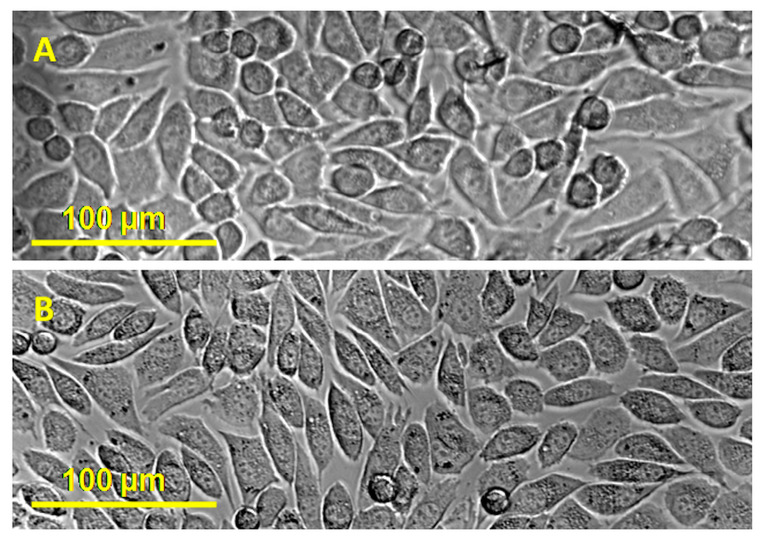
Cytotoxic effect of unmodified nanocellulose (NC/BE 4/0) (**A**) and NC/BE 4/4 (**B**) on the L929 fibroblast cell line.

The images recorded following the cytotoxicity tests for the unmodified NC (NC/BE 4/0) and the NC with the highest amount of BE (NC/BE 4/4) are shown in Figure 11. One can observe that the cells developed normally in the presence of pure nanocellulose, an already established safe material [59], as well as in the presence of the sample containing the maximum amount of BE. Other tests followed the effect of basil extract on SH-SY5Y cells and revealed that the integrity of the cell’s membrane decreased with the addition of basil extract at a 2 mg/mL concentration [60]. In another work, it was observed that basil extracts as infusion (600 mg in 200 mL of distilled water) and as hydroethanolic extract (80:20 *v*/*v*) did not inhibit the normal growth of non-tumoral cells, while the growth of most of the tumoral cells was inhibited [61].

## 3. Materials and Methods

### 3.1. Materials

Microcrystalline cellulose (powder, 20 µm mean particle size) was acquired from Sigma Aldrich (St. Louis, MO, USA). A nanocellulosic suspension (Figure S3—Supplementary Material) was obtained by high-pressure homogenization of a 1.5 wt% MCC aqueous suspension using a microfluidizer (Microfluidics, Westwood, MA, USA) in fifteen consecutive passes. Basil ethanolic extract (BE) (20 g of dried plant mass and 70% *v*/*v* ethanol) was purchased from Hofigal (Bucharest, Romania) and was used unaltered. Basil seeds were purchased from Agrosel Home Gardening (Bucharest, Romania).

### 3.2. Basil Seed Mucilage (BSM) Extraction

A quantity of 2 g of basil seeds was immersed in 135 mL of distilled water and kept at pH = 8 and 57 °C under magnetic stirring for 3 h. The pH of the extraction medium was adjusted using NaOH and HCl solutions with a 0.2 M concentration. The mucilage was separated from the seeds by filtration through a mesh.

### 3.3. Preparation of Nanocellulose Sponges

The nanocellulose suspensions with different contents of basil extract (Table 3) were kept under magnetic stirring (600 rpm) for 10 h at room temperature for optimal impregnation and then freeze-dried for 72 h using a FreeZone 2.5 L Benchtop Freeze Dry System (Laboconco, Kansas City, MO, USA) in the following conditions: −85 °C, 0.008 mbar. The same method was employed for obtaining the sponges containing the mucilage.

### 3.4. Characterization

#### 3.4.1. Fourier-Transform Infrared Spectroscopy (FT-IR) Spectroscopy

FT-IR analysis was carried out on the unmodified nanocellulose sponge and the sponges containing BE or both BE and BSM. For the measurements, a Jasco FTIR 6300 spectrometer (Jasco Co., Tokyo, Japan) was employed and used in the 4000–500 cm^−1^ region employing the attenuated total reflectance (ATR) technique with 32 scans at a resolution of 4 cm^−1^. All FT-IR spectra were normalized at 1060 cm^−1^, corresponding to the C–O stretching vibration [62].

#### 3.4.2. Thermogravimetric Analysis (TGA)

Thermogravimetric analysis, used to characterize the thermal behavior of the sponges, was carried out using a TGA Q5000 from TA Instruments (New Castle, DE, USA) under nitrogen flow of 40 mL min^−1^ with 10 °C min^−1^ temperature advance in the 30–700 °C range.

#### 3.4.3. Scanning Electron Microscopy (SEM)

For the morphological characterization of the sponges, a Hitachi TM4000 plus microscope (Hitachi, Tokyo, Japan) at an accelerating voltage of 15 kV and an 8.5–9.8 mm distance from the samples in the BSE mode was used. Before the analysis, the sponges were sputter-coated with a 5 nm gold layer for better image quality using a Q150R Plus (Quorum Technologies, SXE, Lewes, UK).

#### 3.4.4. Microcomputer Tomography (Micro-CT)

The micro-CT examination of the sponges was conducted using the high-resolution Bruker micro-computer tomograph 1272 instrument (Kontich, Belgium) without any filter. A 50 kV source voltage, a 200 µA current intensity, and a 1000 ms exposure per frame were used for the scanning. The samples were rotated 180 degrees during a 0.2° rotation step that was used throughout the scanning process. The image was reconstructed by averaging 3 frame acquisitions for each unique slice. The image pixel size (scanning resolution) was adjusted to 11 µm and the 2D projections had a resolution of 2452 by 1640 pixels. The 2D radiography dataset was used to create tomograms using the Bruker NRecon 1.7.1.6 tool (Kontich, Belgium). CTVox 3.3.0 r1403 (Bruker, Kontich, Belgium) was used to display the reconstructed tomograms, and CTAn 1.17.7.2 (Bruker, Kontich, Belgium) was used to analyze the samples. In CTAn, quantitative analysis was performed using the image-pixel-size value to measure the surface and volume of the samples.

#### 3.4.5. Dynamic Mechanical Analysis (DMA)

To analyze the mechanical properties of the sponges, a DMA Q800 dynamic mechanical analyzer from TA Instruments (New Castle, DE, USA) was used in compression mode. Sponges of a cylindrical shape with a thickness × diameter of 10 mm × 35 mm were measured with a ramp force of 0.5 N/min up to a maximum force of 18 N. For the calculation of the specific compression strength, the apparent density of the sponges was calculated in accordance with the standard formula (mass divided by volume) in accordance with the ISO 845 standard.

#### 3.4.6. X-ray Diffraction (XRD)

The crystalline structure of nanocellulose sponges was analyzed using the Rigaku SmartLab X-ray diffractometer (Japan) and CuKα radiation (wavelength λ_CuKα_ = 1.541 Å) at 45 kV and 200 mA. Measurements were performed in parallel-beam configuration, and data were collected over a diffraction angle range 2θ = 5–40° with a scan speed of 2°/min.

#### 3.4.7. Antibacterial Activity

Screening of antibacterial activity was performed using the disc diffusion method against *Staphylococcus aureus* ATCC 29213 and *Escherichia coli* ATCC 25922. The NC/BE, NC/BE 4/3, NC/BE 4/4, and NC/BE/BSM 4/3/2 sponges were tested along with a negative control represented by untreated nanocellulose (NC/BE 4/0). Fragments of approximately the same size were cut from the sponges, then placed in Petri dishes and sterilized under UV light for 15 min. *S. aureus* ATCC 29213 and *E. coli* ATCC 25922 bacterial inoculums were prepared in saline physiological water and calibrated to the 0.5 McFarland standard density, corresponding to 1.5–2 × 10^8^ CFU/mL. Mueller–Hinton agar plates were inoculated with a lawn culture using a sterile cotton swab dipped into the inoculum with excess medium removed. Each sample was tested in duplicate, with a negative control (untreated nanocellulose) placed on a plate along with the samples.

#### 3.4.8. Cytotoxicity Tests

The cytotoxicity of the sponge with the maximum tested BE content (NC/BE 4/4) was evaluated against L929 murine fibroblast cell lines and compared to the behavior of the untreated nanocellulose sponge (NC/BE 4/0). The cells were grown in DMEM media (Dulbecco’s Modified Eagle Medium, Lonza, Verviers, Belgium) supplemented with 10% fetal bovine serum (FBS, Biochrom AG, Berlin, Germany), 100 U/mL penicillin, and 100 μg/mL streptomycin (Lonza, Verviers, Belgium) at 37 °C and 5% CO_2_ in a humid atmosphere. The samples were handled in a sterile environment, and a portion of every sponge was laid on 96-well plates. A quantity of 200 μL of media with 5 · 10^4^ cells/mL was poured over the samples and then incubated for 48 h. An inverted microscope, the Nikon T 2000, was used to capture images.

## 4. Conclusions

In this work, basil ethanolic extract (BE) and basil seed mucilage (BSM) were used as antibacterial agents in nanocellulose. The NC/BE and NC/BE/BSM sponges were obtained by adding different amounts of BE and BSM to nanocellulose suspensions after freeze-drying. The highly volatile active compounds in BE led to a slight decrease in the onset degradation temperature of the NC/BE sponges. The presence of BE or BE/BSM did not change the opened-cell structure and nanofibrillar morphology of the NC sponges but led to highly impregnated cellulose nanofibers with an effect on the open porosity and the pore’s size. The addition of BE increased the specific compression strength of the NC/BE sponges, with a higher amount of BE having a stronger effect. A slight inhibition of *S. aureus* growth was observed in the NC/BE sponges with a higher amount of BE, and no effect was observed in the unmodified NC. In addition, the NC/BE sponge with the highest amount of BE and the best antibacterial effect showed no cytotoxic effect, similar to the unmodified NC. This work proposes a simple method to obtain highly porous nanocellulose structures containing antibacterial basil extract for biomedical applications.

## Figures and Tables

**Table 1:** Characteristic temperatures and residue at 600 °C for the NC/BE and NC/BE/BSM sets, as well as for the pristine NC.

Sample	T_5%_ (°C)	T_on_ (°C)	T_max_ (°C)	R_600 °C_ (%)
NC/BE 4/0	219.2	315.5	335.6	2.6
NC/BE 4/2	208.8	302.4	337.3	6.2
NC/BE 4/3	208.8	300.1	333.4	6.8
NC/BE 4/4	210.7	298.8	332.1	8.7
NC/BE/BSM 4/3/1	215.3	291.7	327.7	4.7
NC/BE/BSM 4/3/2	209.8	289.9	328.5	5.1

**Table 2:** Apparent density and compression strength at 50% strain for the NC/BE sponges.

Sample	Apparent Density (mg cm^−3^)	Compression Strength at 50% Strain (kPa)
NC/BE 4/0	17	0.95
NC/BE 4/2	17	1.69
NC/BE 4/3	16	1.92
NC/BE 4/4	18	2.78
NC/BE/BSM 4/3/1	14	2.21
NC/BE/BSM 4/3/2	14	1.85

**Table 3:** The composition of the nanocellulose formulations.

Sample	NC Suspension (g)	Basil Extract (g)	Basil Seed Mucilage (g)
NC/BE 4/0	50	-	-
NC/BE 4/2	50	1.875	-
NC/BE 4/3	50	2.813	-
NC/BE 4/4	50	3.750	-
NC/BE/BSM 4/3/1	50	2.813	12.5
NC/BE/BSM 4/3/2	50	2.813	25.0

## Data Availability

The data presented in this study are available on request from the corresponding author.

## Supplementary Materials

The following supporting information can be downloaded at: https://www.mdpi.com/article/10.3390/ijms241411871/s1.
