# Supplementary material for: Nanocellulose Sponges Containing Antibacterial Basil Extract
Source: Int J Mol Sci. 2023 Jul 24;24(14):11871. doi: 10.3390/ijms241411871 (PMC10380770; doi:10.3390/ijms241411871)
Supplement: Supplementary file 1 [file ijms-24-11871-s001.zip › ijms-2510024-supplementary.pdf]

Supplementary Material

Nanocellulose sponges containing antibacterial basil extract

Gabriela Mădălina Oprică [1], Denis Mihaela Panaitescu [1,*], Catalina Diana Usurelu [1,2], George Mihai Vlăsceanu [2], Paul Octavian Stanescu [2], Brandusa Elena Lixandru [3], Valentin Vasile [3], Augusta Raluca Gabor [1], Cristian-Andi Nicolae [1], Marius Ghiurea [1] and Adriana Nicoleta Frone [1,*]

[1] National Institute for Research and Development in Chemistry and Petrochemistry, 202 Spl. Independentei, 060021 Bucharest, Romania; madalina.oprica@icechim.ro (G.M.O.); catalina.usurelu@icechim.ro (C.D.U.); raluca.gabor@icechim.ro (A.R.G.); cristian.nicolae@icechim.ro (C.-A.N.); ghiurea@gmail.com (M.G.)

[2] Faculty of Chemical Engineering and Biotechnology, University Politehnica of Bucharest, 1-7 Gh. Polizu Street, 011061 Bucharest, Romania; george.vlasceanu@upb.ro (G.M.V.); paul.stanescu@upb.ro (P.O.S.)

[3] Cantacuzino National Medical-Military Institute for Research and Development, 103 Spl. Independentei, 050096 Bucharest, Romania; brandusa_lixandru@yahoo.com (B.E.L.); vali909.g@gmail.com (V.V.)

* Correspondence: panaitescu@icechim.ro (D.M.P.); adriana.frone@icechim.ro (A.N.F.)

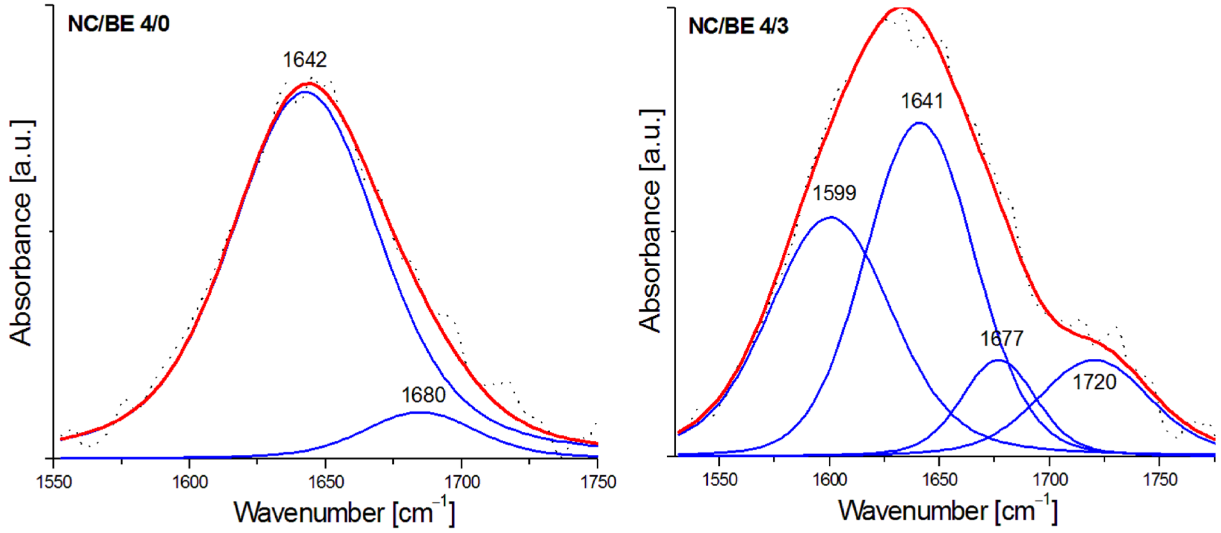

Figure S1. Deconvoluted FT-IR spectra of NC/BE 4/0 and NC/BE 4/3 in the region of 1750–1550 cm^{-1}

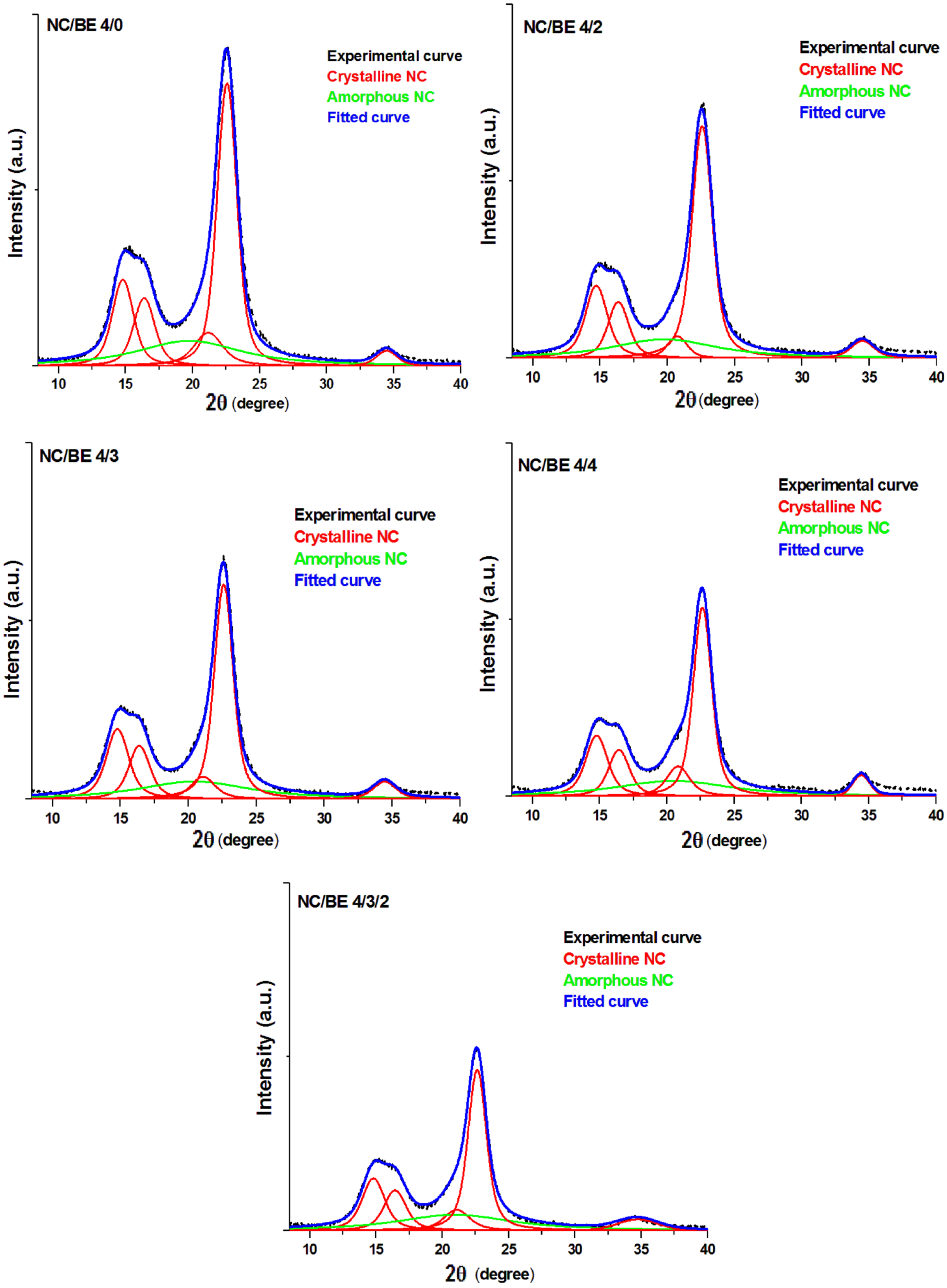

Figure S2. Deconvoluted XRD patterns for the NC sponges containing BE

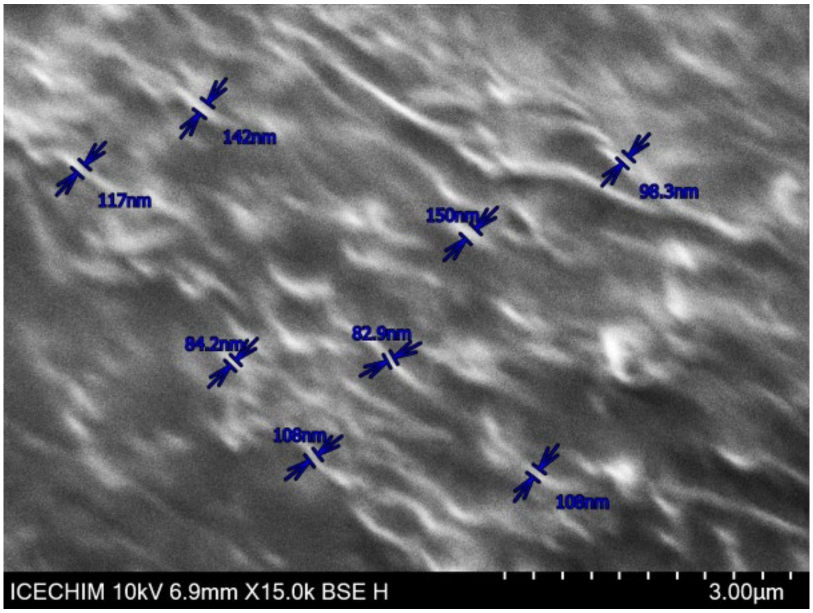

Figure S3. SEM image of nanocellulose casted from water suspension
